# Characteristics of Dialdehyde Cellulose Nanofibrils Derived from Cotton Linter Fibers and Wood Fibers

**DOI:** 10.3390/molecules29071664

**Published:** 2024-04-07

**Authors:** Qiyuan Tu, Wenhua Gao, Junjie Zhou, Jinglin Wu, Jinsong Zeng, Bin Wang, Jun Xu

**Affiliations:** 1 State Key Laboratory of Pulp and Paper Engineering, South China University of Technology, Guangzhou 510640, China; 202120128958@mail.scut.edu.cn (Q.T.); 202220128080@mail.scut.edu.cn (J.Z.); 202221029034@mail.scut.edu.cn (J.W.); fezengjs@scut.edu.cn (J.Z.); febwang@scut.edu.cn (B.W.); xujun@scut.edu.cn (J.X.); 2 Guangdong Provincial Key Laboratory of Plant Resources Biorefinery, Guangzhou 510006, China

**Keywords:** cotton linter fibers, periodate oxidation, dialdehyde cellulose nanofibrils, concentration process, film

## Abstract

Two types of cellulose nanofibrils (CNFs) were isolated from cotton linter fibers and hardwood fibers through mechanical fibrillation methods. The dialdehyde cellulose nanofibrils (DACNFs) were prepared through the periodate oxidation method, and their morphological and structural properties were investigated. The characteristics of the DACNFs during the concentration process were also explored. The AFM analysis results showed that the mean diameters of wood fiber-based CNFs and cotton fiber-based CNFs were about 52.03 nm and 69.51 nm, respectively. However, the periodate oxidation treatment process obviously reduced the nanofibril size and destroyed the crystalline region of the nanofibrils. Due to the high crystallinity of cotton fibers, the cotton fiber-based DACNFs exhibited a lower aldehyde content and suspension stability compared to the wood fiber-based DACNFs. For the concentration process of the DACNF suspension, the bound water content of the concentrated cotton fiber-based DACNFs was lowered to 0.41 g/g, which indicated that the cotton fiber-based DACNFs could have good redispersibility. Both the wood fiber-based and cotton fiber-based DACNF films showed relatively good transmittance and mechanical strength. In addition, to the cotton fiber-based DACNF films had a very low swelling ratio, and the barrier water vapor and oxygen properties of the redispersed cotton fiber-based DACNF films decreased by very little. In sum, this study has demonstrated that cotton fibers could serve as an effective alternative to wood fibers for preparing CNFs, and that cotton fiber-based DACNFs have huge application prospects in the field of packaging film materials due to their stable properties during the concentration process.

## 1. Introduction

Lignocellulosic biomass, which primarily consists of cellulose, hemicellulose, and lignin, has been recognized as a potentially valuable raw material for producing renewable energies and chemicals to replace fossil products [1]. In the high-value utilization of lignocellulosic biomass, in addition to using the sugar platform to undergo bioconversion [2], an important approach is to take advantage of the cellulose platform for the conversion of nanocellulose. Cellulose nanofibrils (CNFs) are nanomaterials that are separated from cellulosic fibers. They not only achieve the characteristics of cellulose, including non-toxic, renewable, and decomposable properties, but also possesses their unique characteristics, such as a large specific surface area, small fiber size, and high aspect ratio. The main fiber raw materials for CNFs include wood fiber, grass and plant fibers, and agricultural waste, which need to be separated and purified through relevant processes that require a significant consumption of chemicals and energy [3,4,5,6,7]. 

Cotton stands as a premier natural fiber crop globally; it has been planted in over 80 countries and is expected to reach an annual production of 30.6 million tons by 2031 [8]. Compared with wood fiber production, the process of cotton linter fiber production is simpler, requiring less energy and fewer chemical agents. Furthermore, cotton linter fibers possess an ultra-high cellulose content (approximately 90~95%), making them an ideal raw material for preparing CNFs [9]. However, numerous studies have focused on prepared, short, rod-like CNCs obtained through the use of strong acids like H_2_SO_4_, H_3_PO_4_, and HCl, etc., on cotton fibers [10]. However, there is a scarcity of research specifically addressing high-aspect-ratio CNFs from cotton linter fibers.

CNFs can also be further chemically modified to impart them with new characteristics. Periodate oxidation can cleave the carbon–carbon bonds at the C2 and C3 positions of cellulose and convert the vicinal hydroxyl groups to two aldehyde groups [11,12]. Dialdehyde cellulose nanofibrils (DACNFs) are an important CNFs derivative. Their aldehyde groups can be modified to carboxylic groups, imines, dialcohol, etc., owing to their high reactivity. This flexible characteristic makes dialdehyde cellulose widely used in various fields, including the adsorption of dyes and metal ions, protein immobilization, drug delivery, wastewater, and sensors [13,14,15,16]. In addition, periodate is not only less toxic but also cost-effective due to its recyclability [17]. Therefore, preparing functionalized CNF materials via periodate oxidation is a feasible strategy that can be conducive to enhancing the application value of CNF products.

Generally, CNF suspensions have a relatively low solid content due to their hydrophilic nature and gel-like behavior [18]. Therefore, the drying and dewatering processes are essential for decreasing transportation and storage costs in commercial production. However, research has demonstrated that this processing procedure has the potential to change the characteristics of CNFs. Wang et al. have observed that the concentration process led to an increase in the average diameter of LCNFs, altering the performance of the films [19]. Moreover, the drying process also has a significant effect on the crystal width of cotton fibers [20]. The work of Liu et al. has proved that the concentration process not only affected the morphological structure and film performance of CNFs but also caused the formation of pores between fibrils [21]. Unfortunately, they did not explore variations in the properties of different cellulose sources during the concentration process. In general, the concentration process inevitably affects the properties of CNF products. Considering the differences in properties between cotton linter fibers and wood fibers, the concentration process may have different effects on the morphological and structural characteristics of cotton fiber-based DACNFs. Moreover, there is also a lack of research on the effect of periodate oxidation on the concentration characteristics of CNFs because research on periodate-oxidized nanocellulose has predominantly focused on assessing the influence of its degree of chemical modification or on enhancing composite properties. Consequently, there is a need for a more comprehensive exploration of this aspect.

Relatively few studies have been conducted on cotton fiber-based CNFs and their derivative DACNFs. Considering the high-cost problems associated with the actual transport of CNF and DACNF products, studies on the concentration and dewatering of CNFs and their derivative products from different feedstocks have not yet been fully developed. Therefore, the aim of this study was to compare the property changes of cotton fiber-based CNFs and wood fiber-based CNFs after periodate oxidation, and to investigate the properties of DACNFs from different feedstocks during the process of concentration and dewatering, as well as in the performance of films prepared after redispersion. 

## 2. Results and Discussion 

### 2.1. Chemical and Physical Properties of DACNFs

**Figure 1:**
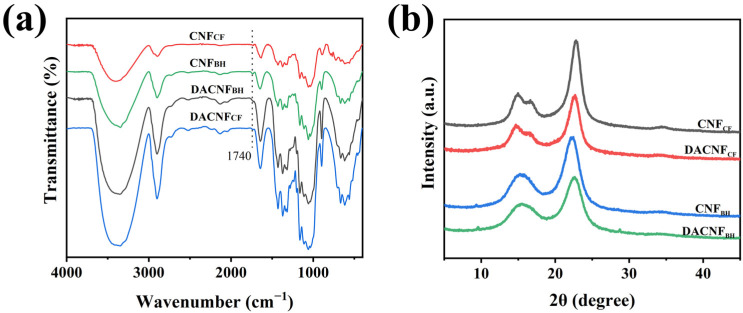
(**a**) FTIR spectra and (**b**) XRD spectra of cotton fiber-based CNFs, DACNFs and wood fiber-based CNFs, DACNFs.

As shown in Figure 1a, the peak at 3400 cm^−1^ corresponding to the stretching vibration of -OH was observed in the spectra of the CNFs and DACNFs. The hydroxyl strength of the single bond in the DACNFs decreased compared with that in the CNFs. The characteristic peaks at 1735 cm^−1^ were attributed to the stretching vibration of aldehyde groups [22]. Its appearance in the spectra of DACNF_CF_ and DACNF_BH_ prove that the hydroxyl group in the CNFs had been successfully oxidized to an aldehyde group. Furthermore, at the same oxidant concentration and reaction time, the aldehyde group content of DACNF_BH_ was 0.93 mmol·g^−1^, which was higher than that of DACNF_CF_ (0.82 mmol·g^−1^). This indicates a relatively high degree of oxidation for DACNF_BH_.

The extent of crystallization of the CNFs is shown to significantly impact physical and chemical properties, such as strength, water retention value, and chemical reactivity [23,24]. To investigate the changes in the crystal structure of the DACNFs, XRD tests were performed. As showed in Figure 1b, all the samples exhibited essentially the same diffraction peaks at 2θ = 15.1°, 16.5°, and 22.5°, corresponding to the (110), (110), and (200) crystal planes of cellulose I, respectively [25]. This confirms that the introduction of aldehyde groups did not alter the crystal structure of the CNFs. Table 1 illustrates the crystallinity and crystallite size of the CNFs, DACNFs, and redispersed DACNFs. The crystallinity of CNF_CF_ was 73.44, which was consistent with the result of the CNC extracted from waste cotton fibers by Cao et al. [26]. CNF_CF_ showed a higher crystallinity and crystallite size than CNF_BH_. This difference could be attributed to the highly ordered crystal structure and natural large cellulose crystal size in CNF_CF_. Another possible explanation is the presence of amorphous hemicellulose and lignin in CNF_BH_. After the same oxidation and homogenization treatments, the crystallinity and crystallite size of the DACNFs decreased due to the destruction of the ordered cellulose structure and the opening of glucose unit rings [16]. This result aligns with the data reported in the literature [27]. The crystallinity of DACNF_CF_ decreased by 4.23%, while that of DACNF_BH_ decreased by 13.40%, indicating that CNF_BH_ was more sensitive to oxidation and homogenization. Additionally, the crystallinity changes in DACNF_CF_ and DACNF_BH_ were all below 0.6% after concentration and redispersion, suggesting that the concentration process had a negligible impact on the crystal structure of the DACNFs. Similar results have been reported in previous studies [19,21].

Table 2 shows the chemical compositions of the cotton linter pulp and BHKP. The cellulose content of the cotton linter pulp was 94.15%, significantly surpassing the cellulose content of the BHKP (74.70%). The hemicellulose content of the BHKP was 18.7% which was higher than that of the cotton linter pulp. The relatively high hemicellulose content in the BHKP was beneficial for fiber swelling and softening prior to the mechanical treatment, facilitating easier nanofibrillation during the homogenization process [28].

The additional properties of the CNFs, DACNFs, and D-DACNFs are illustrated in Table 1. The water retention value (WRV) is a crucial parameter reflecting the extent of fibrillation of CNFs [29]. The WRV of CNF_CF_ was 311.38%, which was lower than that of CNF_BH_ (427.95%), indicating a more noticeable degree of nanofibrillation in CNF_BH_. After oxidation and homogenization, the WRV of DACNF_CF_ and DACNF_BH_ increased by 16.36% and 21.78%, respectively. The was due to an increase in the available surface area of the fibrils, providing more binding sites for water molecules. The specific surface area (SSA) of the DACNFs showed a similar trend to that observed in the WRV. The increase in SSA in the DACNFs was mainly due to the reduction in diameter size by oxidation and homogenization, which increased the number of active hydroxyl groups capable of adsorbing Congo Red. The CNFs and DACNFs obtained from hardwood fibers showed relatively high SSAs, owing to their relatively loose internal structure and comparatively small fibril diameter. Zeta potential can reflect the stability of a sample suspension. As shown in Table 1, DACNF_BH_ demonstrated relatively high zeta potential (−19.84 mV), indicating excellent homogeneity and dispersion. In addition, the DACNFs showed low surface charge densities, which are associated with the introduction of aldehyde groups during the oxidation process. After the concentration and redispersion, there was a notable reduction in the WRV, SSA, zeta potential, and surface charge density of the D-DACNFs. The observed phenomenon could be explained by the agglomeration of fibrils in the DACNFs as a resulted of the formation of irreversible hydrogen bonding during the concentration process [30]. This made them challenging to disperse completely under high shear forces. The agglomeration of fibrils not only led to a reduction in exposed hydroxyl groups but also contributed to an increase in fibrils size. Consequently, this resulted in a decline in properties such as WRV, SSA, surface charge density, and the absolute value of the zeta potential of the D-DACNFs. Interestingly, D-DACNF_BH_ showed a more significant reduction in the same properties than D-DACNF_CF_. For example, after redispersion, the SSA of D-DACNF_CF_ decreased by 23.06%, while D-DACNF_BH_ decreased by 75.78%. The poorer redispersibility of D-DACNF_BH_ compared with D-DACNF_CF_ may be attributed to the stronger agglomeration caused by its relatively high aldehyde group content [31]. In addition, its relatively low crystallinity and loose structure led to an increase in the exposed free hydroxyl groups. This resulted in the formation of more irreversible hydrogen bonds between the fibrils during the concentration process, increasing the degree of agglomeration of C-DACNF_BH_. These factors influence its redispersibility and lead to poorer performance. In sum, D-DACNF_CF_ still had relatively favorable properties after concentration and mechanical dispersing. Furthermore, for the DACNF_BH_ with a dewatering degree of 20.0 wt% in this work, the SSA and WRV were reduced by 75.78% and 9.24% after redispersion, respectively. In a previous study using unmodified wood fiber-based CNFs, the SSA and WRV decreased by 5.71% and 3.35%, respectively [21], after redispersion under the same concentration conditions. Therefore, it could be concluded that the redispersibility of the DACNFs was poorer than that of the CNFs. This may be due to the involvement of double aldehyde groups in the formation of irreversible hemiacetal bonds, which makes the DACNFs themselves more prone to aggregation [31]. In addition, the aldehyde groups in the DACNFs were more likely than the hydroxyl groups to form irreversible hydrogen bonds between the fibrils during the dewatering process, thus reducing their redispersibility.

### 2.2. Morphological Structure of CNFs, DACNFs, and Redispersed DACNFs

**Figure 2:**
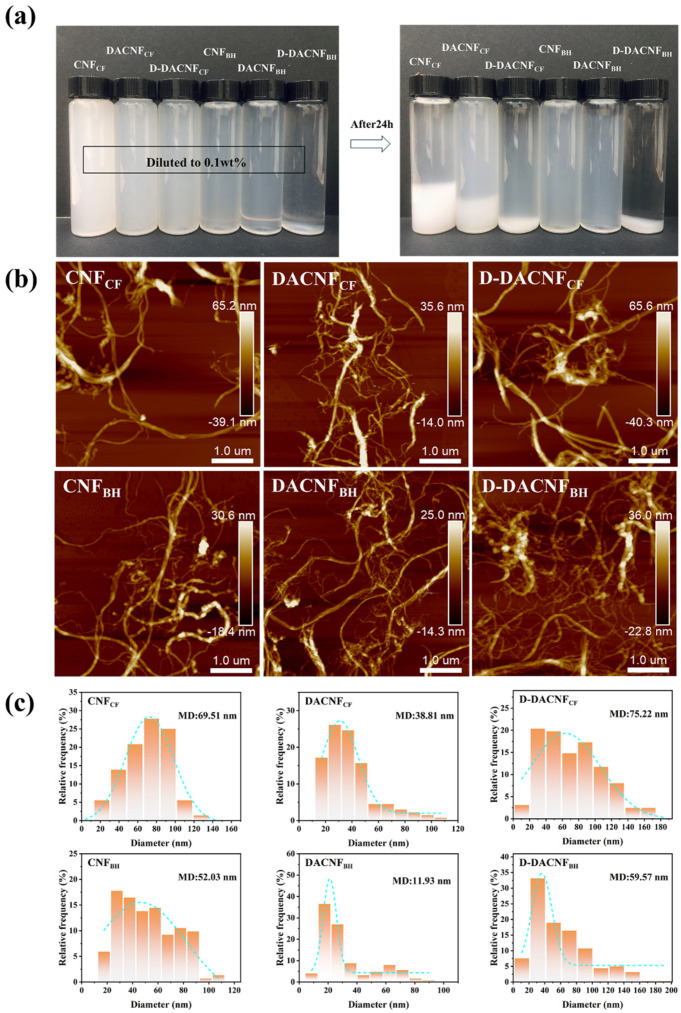
(**a**) Suspension stability, (**b**) AFM images, and (**c**) diameter distribution of cotton fiber-based CNFs, DACNFs, redispersed DACNFs and wood fiber-based CNFs, DACNFs, redispersed DACNFs.

Figure 2a shows the stability of the CNF, DACNF, and D-DACNF suspensions. The different samples at the same concentration displayed varying degrees of sedimentation after 24 h. Little sedimentation was observed in the CNF_BH_ and DACNF_BH_ suspensions, indicating their excellent stability. However, the CNF_CF_ suspensions showed low stability, primarily because the strong hydrogen bonding between the cotton linter fibers hindered their easy separation into nanofibrils during the grinding process [32]. As a result, the final CNF_CF_ contained large fibrils, making it prone to sedimentation. This was supported by the subsequent AFM analysis (Figure 2b,c). Moreover, the stability of the DACNFs was slightly reduced after periodate oxidation, which could be attributed to the introduction of the aldehyde group disrupting the original electrostatic repulsion effect on the fibrils’ surfaces [33]. This was proved by the result on the surface charge density, as shown in Table 2. The further decrease in the stability of the redispersed DACNF suspensions could be attributed to the fibril agglomerations in the C-DACNFs during the concentration process. Apparently, the degree of sedimentation in the D-DACNF_BH_ suspension was relatively higher than that in the D-DACNF_CF_ suspension, indicating that more severe fibril agglomerations occurred in the concentrated DACNF_BH_. One possible reason could be the relatively higher content of aldehyde groups in DACNF_BH_, leading to more fibril agglomeration based on hemiacetal cross-linking formation [31].

The AFM images were used to explore the morphological characteristics of the two DACNFs and their changes during the concentration process. Figure 2b,c shows the morphology and diameter distribution of the different samples. After mechanical grinding, the diameter of both CNF_CF_ and CNF_BH_ were reduced to the nanoscale. However, CNF_BH_ predominantly consisted of individual fibrils, while bundle-like fibrils were abundant in CNF_CF_. This indicates that the cotton linter fiber was more challenging to nanofibrillation than the wood fiber. This difference could be attributed not only to variations in cellulose microfibril widths within the cell walls of the cotton linter fibers and wood fibers but also to disparities in their crystallinity and crystal structure [34,35]. In addition, the delignification process during the bleaching treatment enhanced the mechanical fibrillation capability of the wood fibers [36]. After oxidation and homogenization, a significant reduction in the mean diameter (MD) of the DACNFs was observed, accompanied by a narrowed diameter distribution. This phenomenon was ascribed to the oxidative degradation of cellulose molecular chains [16,37]. It was worth noting that the MD of DACNF_CF_ decreased from 69.51 nm to 38.81 nm, while that of DACNF_BH_ decreased from 52.03 nm to 11.93 nm. The reduction in the MD of DACNF_CF_ was relatively small due to its high crystallinity and crystal size, which made the cell wall structure less susceptible to disintegration. Additionally, the lateral dimensions of the redispersed DACNFs increased, and the diameter distribution exhibited unevenness, as seen in the AFM images and the distribution of diameter. This phenomenon could be explained by the removal of water from the DACNF suspension during the concentration process, leading to significant agglomerations of the fibrils based on hydrogen bonding [38]. These agglomerations were found to be irreversible even after dispersion, resulting in an increase in the lateral dimensions of D-DACNFs. It could be observed that the DACNFs were more susceptible to agglomeration after air-drying compared with the CNFs [16]. This may have been caused by the formation of hydrogen bonds between the aldehyde group and the hydroxyl group on the molecular chain of the DACNFs during the drying process. The above results agree with that observed in the suspension stability investigation (Figure 2a).

### 2.3. Bound Water and Pore Size Distribution of Concentrated CNFs and DACNFs 

**Figure 3:**
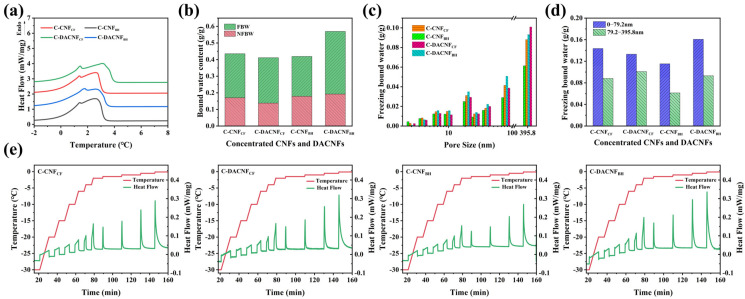
Continuous melting of C-CNFs and C-DACNFs measured by DSC: (**a**) the endothermic melting curves and (**b**) BW content in C-CNFs and C-DACNFs. Isothermal step melting of C-CNFs and C-DACNFs determined by DSC: (**c**) the FBW content of various pores, (**d**) the overall FBW content within pores categorized by sizes of 0–79.2 nm and 79.2–395.8 nm, and (**e**) the heat flow/temperature curve of C-CNFs and C-DACNFs.

**Figure 4:**
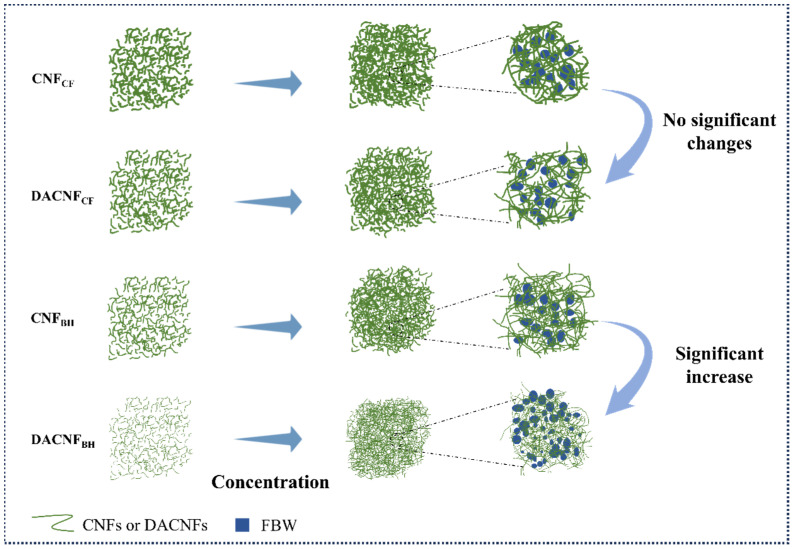
The distribution of CNFs and DACNFs after concentration and the change in FBW content in the pores formed between fibrils during concentration process.

To investigate the characteristics in concentrated the CNFs and DACNFs, the DSC test was utilized. Since CNFs are a porous hydrophilic material, their adsorbed water can be divided into free water and bound water (BW), where bound water equals freezing bound water (FBW) plus non-freezing bound water (NFBW) [39,40,41]. Figure 3a shows the endothermic melting curves of the concentrated CNFs and DACNFs under the DSC continuous melting method. Previous studies have shown that the pores of unconcentrated CNF suspensions were not detected by DSC analysis, owing to their uniform nanoscopic size as well as their stable gel properties [21]. All of them showed FBW melting peaks near 1–2 °C, which denoted the existence of pores in both the C-CNFs and C-DACNFs. These pores were formed due to the agglomeration of fibrils driven by hydrogen bonds and capillary forces, which could efficiently entrap a substantial amount of BW within the nanofibril network. As shown in Figure 3b, the BW contents of C-CNF_CF_, C-DACNF_CF_, and C-CNF_BH_ were calculated to be approximately 0.44 g/g, 0.41 g/g, and 0.42 g/g, respectively, which was consistent with the BW content of the wood fiber-based CNFs (0.38 g/g) reported by Liu et al. [15]. For C-DACNF_BH_, the BW content was determined to be about 0.57 g/g, and this result was nearly twice as high as the content of BW for wood fibers (0.28 g/g) [20]. The BW content in the C-CNF_BH_ and C-CNF_CF_ samples showed little difference, but a notable distinction emerged after the oxidative homogenization. In other words, the fibrils in DACNF_BH_ formed more pores during the concentration process compared with C-CNF_BH,_ while the pores formed in the DACNF_CF_ fibrils were similar to those of C-CNF_CF_ during the concentration process. Figure 4 shows the distribution of CNF_CF_, DACNF_CF,_ CNF_BH_, and DACNF_BH_ and the change in FBW content in the pores formed between fibrils during the concentration process. It has been reported that BW content is closely associated with the size of fibrils and crystallinity [40]. In this work, the loose amorphous zone and small fibrils size of DACNF_BH_ allowed for a great number of hydroxyl groups to be accessible to individual water molecules. Additionally, owing to the relatively high aldehyde group content of DACNF_BH_, it was more prone to strong fibril agglomerations based on comparison with the CNFs during the concentration process. Consequently, C-DACNF_BH_ had the highest content of BW. In addition, the NFBW contents showed a negative association with the crystallinity of the samples. For instance, C-DACNF_BH_ had the lowest crystallinity, but its NFBW content was the highest. This was due to the NFBW content affecting the molecular rearrangement in the intermediate region between the crystalline and amorphous regions [42]. 

The DSC isothermal step melting procedure was utilized to analyze the pore size distribution of the concentrated CNFs and DACNFs. The principle of pore distribution in the concentrated CNFs and DACNFs was detected by using DSC; it was found that the pressure at the curved interface in the capillary cavities of the concentrated CNFs and DACNFs was lower, resulting in the water in the capillary having a lower melting temperature. Since the drop in melting temperature was inversely proportional to the pore diameter, the pore size distribution of the concentrated CNFs and DACNFs could be analyzed by using the isothermal step melting procedure. The heat flow/temperature curves of the C-CNFs and C-DACNFs under this procedure are shown in Figure 3e. The FBW content in pores of different diameters was calculated by integrating the curves for each melting peak and substituting them into the thermodynamic equations. It should be known that the FBW content calculated by this procedure represented all the water in the C-CNFs and C-DACNFs pores. Due to the precision of the equipment being limited to 0.1 °C, we could only obtain the FBW content distribution in the range of 0–395.8 nm, as shown in Figure 3c. During the concentration process, the C-CNFs and C-DACNFs exhibited the formation of pores with varying sizes, and the distribution of these pores was non-uniform. The FBW content in each corresponding pore in the C-DACNFs increased after periodate oxidation and homogenization. The increase in the FBW contents varied in different pore size ranges. The FBW content in smaller pores (pore sizes < 9.9 nm) exhibited a gradual increase, while in larger pores (pore sizes > 9.9 nm), the FBW content showed a noticeable increase. For C-DACNF_BH_, the total FBW content increased by only 7.27% in smaller pores (pore sizes < 9.9 nm), while a significant 53.17% increase was observed in larger pores (pore sizes > 9.9 nm). However, this phenomenon was not evident in C-DACNF_CF_. These results suggest that oxidation and homogenization had little influence on the concentration characteristics of DACNF_CF_. As can be seen from Figure 3d, in the C-CNF and C-DACNF samples, the overall content of FBW in the smaller pores (pore sizes < 79.2 nm) was greater than that in the larger pores (pore sizes 79.2–395.8 nm). This might be due to the small size of the concentrated samples, or it might be attributed to the limited precision of the instrument, which did not provide more detailed pore information. 

### 2.4. Properties of Films

To evaluate how the formation of pores during the concentration of DACNFs affects their subsequent applications and the differences in film properties between CNFs and DACNFs, the properties of the CNF, DACNF, and redispersed DACNF films were examined.

#### 2.4.1. Microstructure of Films

**Figure 5:**
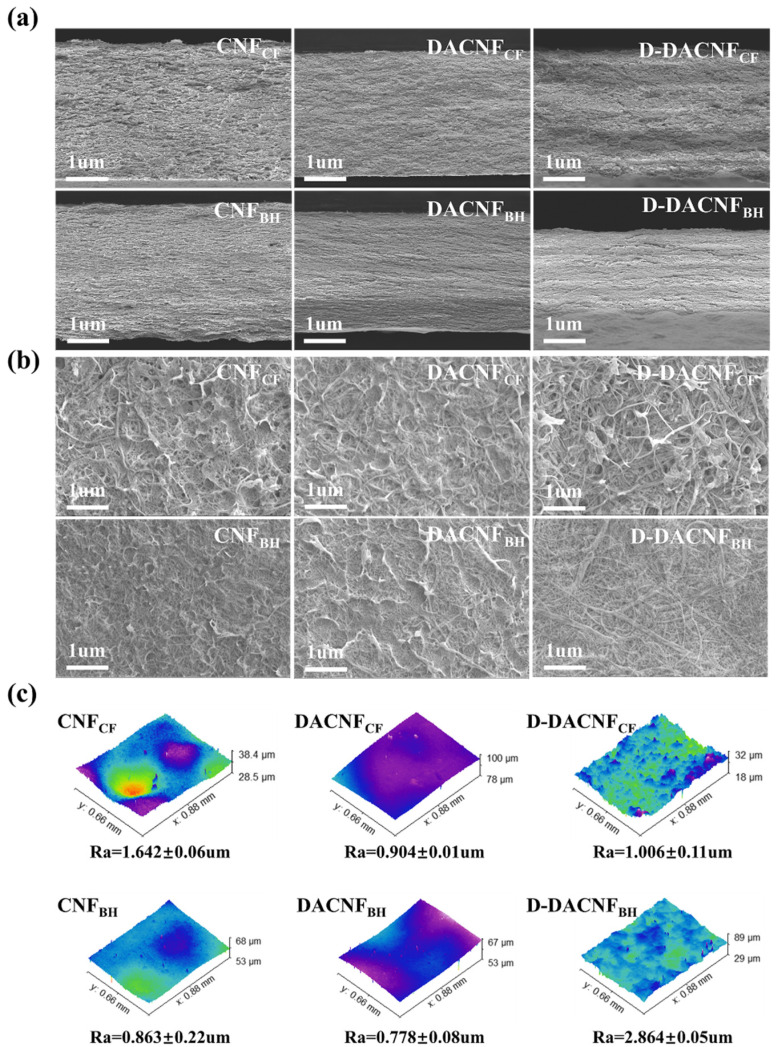
(**a**) The surface morphology, (**b**) the cross-section morphology, and (**c**) the 3D topographies of non-concentrated and redispersed DACNF films.

The surface morphology and fracture cross-section morphology of the films were examined by using SEM. As shown in Figure 5a, the CNF_CF_ film had a relatively rough surface and visible pore structures, indicating that it had a large and uniform diameter distribution. In Figure 5b, in the same basis weight of films, the thickness of the DACNF films was lower than that of the CNF films, indicating that the oxidation and homogenization processes were beneficial for the formation of dense structures in the DACNFs. Due to fibril agglomerations during the concentration process, visible fiber bundles and large pore structures were observed on the surface of the redispersed D-DACNF films, and these features contributed to the increased surface roughness of the films. In addition, some intervals appeared in the cross-sectional of the D-DACNF films, potentially influencing specific properties of the films. As shown in Figure 5c, after redispersion, the roughness change in the D-DACNF_BH_ film was significantly higher than that in the D-DACNF_CF_ film. This again demonstrates that DACNF_CF_ had a relatively stable redispersion performance.

#### 2.4.2. Mechanical Properties of Films 

**Figure 6:**
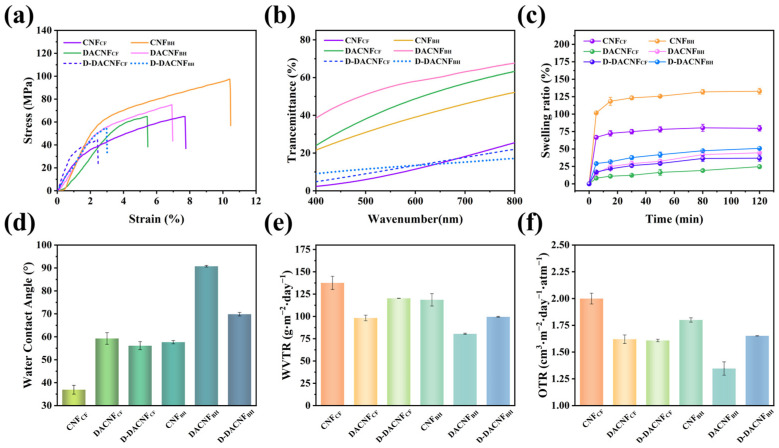
Properties of cotton fiber-based CNF, DACNF, and redispersed DACNF films and wood fiber-based CNF, DACNF, redispersed DACNF films: (**a**) mechanical properties, (**b**) transmittance properties, (**c**) swelling properties, (**d**) water contact angle, (**e**) water vaper transmission rate, and (**f**) oxygen transmission rate.

As depicted in Figure 6a, the tensile strengths of the CNF_CF_ and CNF_BH_ films were 64.85 and 96.97 Mpa, respectively. The results were similar to the film tensile parameters reported by Lin et al. [43]. The tensile strength of the CNF_BH_ film was higher than that of the CNF_CF_ film, which is attributed to the smaller mean diameter of CNF_BH_ leading to the formation of a denser film structure. Furthermore, due to its flexible and fibrillar structure, the CNF_BH_ film exhibited the highest tensile strain value of 10.45%. Periodate oxidation and homogenization resulted in a significant decrease in the tensile strength and elongation of the DACNFs. Similar behavior was previously observed in bacterial cellulose with different degrees of periodate oxidation [44]. After concentration and redispersion, the tensile strength of the D-DACNF_BH_ films reduced by 22.9%, while that of the D-DACNF_CF_ films decreased by only 0.6%. The reason for the decrease in tensile strength could be explained by the presence of fibril agglomerations on the surface of the D-DACNF films. Even with very high mechanical shear force, it was difficult to disperse these aggregated fibrils, leading to a decline in the tensile properties of the film. The difference in the extent of the reduction in tensile properties suggests that DACNF_CF_ had better redispersibility.

#### 2.4.3. Optical Properties of Films 

The light transmittances of the CNF, DACNF, and redispersed DACNF films (30 g/m^2^) at 400–800 nm are shown in Figure 6b. The optical properties were primarily influenced by the MD size and diameter distribution of the CNFs. Consequently, the CNF_CF_ film exhibited a very low light transmittance. Compared with CNF films of the same thickness reported in the literature, the transmittance of the CNF_BH_ film was similar to that of purely mechanically prepared CNFs film from hardwood pulp [21]. The DACNF films showed the highest transmittance due to their small and relatively uniform fibrils size, resulting in a reduced scattering effect of ultraviolet light. The D-DACNF_CF_ and D-DACNF_BH_ films showed low light transmittance due to the pore structure and fibril agglomerations on their film surfaces. After concentration and redispersion, the transmittance of the D-DACNF_CF_ film experienced a reduction of 3.99%, whereas the D-DACNF_BH_ film showed a more significant decrease of 20.12%. This indicates that DACNF_CF_ had a relatively good redispersibility.

#### 2.4.4. Swelling Capacity and Hydrophobicity of Films 

The swelling capacities of the CNF, DACNF, and D-DACNF films are shown in Figure 6c. For all the films, as the immersion time increased, the swelling ratio showed a relative increase, particularly in the initial 5 min. Among all the films, the CNF_BH_ film showed the highest swelling rate, reaching 125%, which is approximately twice that of the CNF_CF_ film. This could be explained by the low crystallinity and relatively high hemicellulose content of CNF_BH_, which allowed it to absorb more water. The oxidation combined with homogenization resulted in a significant decrease in the swelling rate of the CNF films. Specifically, the swelling rates of the CNF_CF_ and CNF_BH_ films decreased by 60.86% and 57.22%, respectively. This was due to the smooth surface and low pore structure of the DACNFs, which generated a strong capillary force on the film surface, impeding the entry of water molecules [45]. Duran et al. observed the same trend in the rate of moisture sorption when introducing aldehyde into CNFs [46]. After 120 min of immersion, the swelling ratio of the D-DACNF films exceeded that of the DACNF films slightly, owing to the relatively rough surfaces and the intervals formed between fibrils. This result was supported by the SEM images and the roughness of films presented in Figure 5. 

The hydrophilic properties of the CNFs, DACNFs, and redispersed DACNFs were also characterized. The water contact angle results for the films are presented in Figure 6d. Both the CNF_CF_ and CNF_BH_ films were hydrophilic because they showed low contact angles. This also indicates their richness in hydroxyl groups. The introduction of an aldehyde group via the periodate oxidation was found to have significant impacts on the hydrophobicity of the films. The water contact angles of the DACNF_CF_ and DACNF_BH_ films were 59.26° and 90.74°, respectively, which are significantly higher than those of CNFs. This was due to the introduced aldehyde groups reducing the number of hydroxyl groups that could interact with water molecules. This result is in agreement with earlier reports that found hydrophobicity increased when the aldehyde group was introduced [47].

#### 2.4.5. Barrier Performance of Films

The barrier properties of the CNF, DACNF, and redispersed DACNF films were assessed by measuring the water vapor transmission rate (WVTR) and oxygen transmission rate (OTR), and the results are presented in Figure 6e,f. Compared with the CNF_CF_ film, the CNF_BH_ film showed a relatively high-water barrier performance and oxygen barrier performance due to its smaller size. After oxidation, the WVTR of the DACNF_CF_ film and the DACNF_BH_ film decreased by 21.80% and 32.14%, respectively. Additionally, the OTR of the DACNF_CF_ film and the DACNF_BH_ film decreased by 19.00% and 25.20%, respectively. The decreases in the WVTR and OTR were attributed to the formation of relatively dense structure in the DACNF films, significantly reducing the permeability to water and oxygen molecules. However, the WVTR and OTR of the D-DACNF films increased after redispersion. This increase was attributed to the irreversible binding of fibrils in the DACNFs during the concentration process, resulting in the D-DACNFs failing to form a uniform morphological structure. This result was corroborated by the SEM images presented in Figure 5. In addition, the change in the D-DACNF_CF_ film barrier properties was smaller than that of the D-DACNF_BH_ film, demonstrating that the concentration process had really little effect on the characteristics of DACNF_CF_, as previously discussed. 

## 3. Experimental

### 3.1. Materials

Bleached hardwood kraft pulp (BHKP) was provided by Jiangsu UPM-kymmene Co., Ltd. (Changshu, China). Cotton linter pulp was kindly provided by the Cotton Research Institute of Academy of Agricultural Sciences (Hefei, China). The composition of the cotton linter pulp and BHKP was tested according to the NREL Technical Report [48]. The sodium periodate, ethylene glycol, sodium hydroxide, and potassium bromide were all of analytically pure grade and were purchased from Shanghai Macklin Biochemical Co., Ltd. (Shanghai, China).

### 3.2. Preparation of Cellulose Nanofibrils (CNFs)

The dry cotton linter pulp and BHKP were torn into small pieces and fully immersed in water for 24 h, respectively. Then, the cotton linter pulp was defibered to a beating degree of 70 °SR by using a valley beater (PL4-2, FFiber, Dongguan, China). After that, the cotton linter pulp and BHKP were diluted to a concentration of 1.0 wt% with distilled water, respectively. After that, the slurry was mechanically fibrillated by using the MKCA6-2J Supermasscolloider (Masuko Sangyo Co., Ltd., Kawaguchi-City, Japan). First, the gap between the grinding discs was sequentially set to +20 μm, 0 μm, and −20 μm, with a speed of 1500 rpm. Second, the gap between the grinding discs was sequentially set to −50 μm, −80 μm, −100 μm, −150 μm, and −180 μm, with a speed of 1500 rpm. At each stage the fiber underwent ten consecutive grinding cycles. For the convenience of subsequent discussion, the CNFs produced from the cotton linter pulp and BHKP were labeled as CNF_CF_ and CNF_BH_, respectively.

### 3.3. Periodate Oxidation of CNFs

The oxidation process of the CNFs (with an absolute dry mass of 15 g) was carried out in a dark environment. The mass ratio of sodium periodate to CNFs was set at 1:1. The pulp consistency was adjusted to 0.5 wt%, and the mixture was heated in a water bath at 45 °C for 4 h. At the end of the reaction, approximately 10 mL of ethylene glycol was introduced to terminate the oxidation process, and stirring was continued for an additional 30 min. The sample was then washed by centrifugation several times at 2000 rpm with deionized water. After that, the samples were ground at 1376 bar 5 times by using a high-pressure homogenizer (JN-100FS, Guangzhou Juneng Nano Bio Technology Co., Ltd., Guangzhou, China) and then stored at 4 °C. The DACNFs obtained from CNF_CF_ and CNF_BH_ were named DACNF_CF_ and DACNF_BH_, respectively. The advantages of unoxidized and dialdehyde cellulose nanofibrils would be verified, and this study did not consider the impact of aldehyde content variations on DACNFs.

### 3.4. Concentration and Redispersion of CNFs and DACNFs

The CNFs and DACNFs obtained from the two raw materials were concentrated to 20 wt% by using an H2050R centrifuge (Hunan Xiangyi Centrifuge Instrument Co., Ltd., Changsha, China), respectively. Specially, the ~1.0 wt% CNF and DACNF suspensions were initially centrifuged at 2000 rpm for 5 min, followed by centrifugation at 6500 rpm for 5 min. The concentrated CNF_CF_, DACNF_CF_, CNF_BH_, and DACNF_BH_ samples were denoted as C-CNF_CF_, C-DACNF_CF_, C-CNF_BH_, and C-DACNF_BH_, respectively. The concentrated DACNF samples were redispersed. First, the concentration of the C-DACNF sample was diluted to the initial concentration (1.0 wt%), respectively. Subsequently, the C-DACNF samples were evenly dispersed using a T 25 digital ULTRA-TURRAX^®^ IKA^®^ high-speed homogenizer (IKA, Nürnberg, Germany) at 10,000 rpm for 10 min. The redispersed DACNF_CF_ and DACNF_BH_ suspensions were labeled as D-DACNF_CF_ and D-DACNF_BH_, respectively.

### 3.5. Preparation of Films 

The CNF films, DACNF films, and D-DACNF films were prepared using the vacuum filtration method. The film basis weight was 50 g/m^2^. The pore size of the filter films used was 0.22 μm. The films were dried for 48 h under the conditions of 5.0 MPa pressure at 30 °C. Finally, the films were allowed to equilibrate in an environment of 22 °C and a relative humidity of 55% for 24 h to achieve moisture content balance.

### 3.6. Characterization of CNFs and DACNFs 

#### 3.6.1. Determination of Aldehyde Content

The initial pH of the hydroxylamine hydrochloride solution (0.25 M) was determined using a pH meter. Then, 25 mL of this solution was pipetted and mixed with the sample (with an absolute dry mass of 0.10 g), and the mixture was stirred at room temperature for 24 h. After that, the solution was filtered using filter paper. The filtrate was titrated with a standard NaOH solution (0.01 M) to bring the pH back to its initial value. The aldehyde group content (mmol·g^−1^) of the DACNF samples was calculated according to Equation (1):

(1) $$n=\frac{C\times V}{M}$$

where *V* refers to the volume of NaOH consumed in the titration (mL), *C* is concentration of NaOH (mol·L^−1^), and *M* is weight of sample (g).

#### 3.6.2. Fourier Transform Infrared Spectroscopy (FTIR)

A certain amount of the sample and KBr powder (Spectro grade, 99.9–100%) were mixed evenly, and the mixture was pressed into thin slices for IR spectroscopy by using a tablet press. The FTIR spectra were recorded on a FTIR spectrometer (Nicolet IS50-Nicolet Continuum, Thermo Fisher Scientific, Waltham, MA, USA) at a resolution of 4 cm^−1^ between 400 and 4000 cm^−1^ and scanned 32 times.

#### 3.6.3. Atomic Force Microscopy (AFM)

The morphology of the CNF, DACNF, and D-DACNF samples was analyzed by using AFM (Leica TCSSP5, Germany). The mass concentration of the samples ranged from 1 × 10^−6^ wt% to 1 × 10^−5^ wt%. The sample suspensions were ultrasonically dispersed at −10 °C for 1 min. After that, a small drop was taken on a mica sheet. After the samples had naturally dried, the morphological characteristics of the sample were observed. The AFM images were captured at room temperature by using an AFM probe (SNL-10, Bruker Inc., Santa Barbara, CA, USA) in the PeakForce™ ScanAssyst mode. The utilized AFM probe had a resonance frequency (f_0_) of 56 kHz and was characterized by a spring constant (k) of 0.24 N/m. The fiber sizes in the AFM images were determined by using Nano Measurer 1.2 software. 

#### 3.6.4. X-ray Diffraction (XRD)

The crystallinity of the CNF, DACNF, and D-DACNF samples was determined by using an XRD diffractometer (D/max-IIIA, Japan). Cu-Kα radiation filtered through nickel was used. The scan rate was 2.0 °/min and the scanning range was between 5° and 60° for 2θ. The crystallinity index (CrI) was calculated according to Equation (2):

(2) $$\mathrm{CrI}=\frac{I_{200}{-I}_{\mathrm{am}}}{I_{200}}\;\times \;100\%$$

where *I*_200_ is the diffraction intensity of the 200 lattice plane at 2*θ* = 22.5°; *I_am_* is the diffraction intensity of the amorphous region; and for cellulose *I* is 2*θ* = 18.0°.

The average crystallite size perpendicular to the 200 lattice plane was determined by using Equation (3) [49]:

(3) $$D_{\mathrm{hkl}}=\frac{0.9\lambda }{\beta \cos\theta }$$

where *k* is the Scherrer constant and λ represents the wavelength of Cu-Kα radiation (0.15418 nm), *β* refers to the width at half height of the 200 lattice plane in radian, and *θ* is the Bragg’s angle in radian, calculated as in the excel spreadsheet.

#### 3.6.5. Specific Surface Area (SSA)

The SSAs of the CNFs, DACNFs, and D-DACNFs were tested according to the Congo Red dye adsorption method [50]. Congo Red (75% of the dry weight of the sample) was added to the samples, and then deionized water was added to adjust the consistency to 0.2 wt%. The mixtures were thoroughly mixed for 24 h at 300 rpm and 60 °C. Subsequently, the mixtures were centrifuged at a speed of 5000 rpm for 20 min. The absorbance of the sample supernatants after centrifugation were measured at 495 nm by using a UV-visible spectrophotometer (UV-2600, Shimadzu, Japan). The Congo Red concentration in the sample supernatants were determined based on the standard curve derived from the Congo Red solutions. The specific surface area (*A_cell_*, m^2^/g) of the samples were calculated from the following Equation (4):

(4) $$A_{\mathrm{cell}\;}=\frac{6.024{\;\times \;10}^{23}\;m_{g}A_{d}}{M_{g}}$$

where *A_d_* represents the area of dye per molecule (1.73 nm^2^), *M_g_* is the relative molecular weight of dye molecules (696.68 g/mol), and *m_g_* signifies the saturation value of sample adsorption (g·kg^−1^).

#### 3.6.6. Water Retention Value (WRV)

The WRVs of the CNF, DACNF, and D-DACNF samples were determined by using the TAPPI standard method [29]. The samples were centrifuged with a centrifugal force of 3000× *g* for 30 min. Then, the samples were dried for 24 h at 105 °C to constant weights. The WRVs were determined by using the ratio of the weight of retained water in the centrifuged sample to the absolute dry mass of the sample.

#### 3.6.7. Zeta Potential

A Malvern nanoparticle analyzer (Zetasizer Ultra, Malvern, UK) was used to measure the seta potential of the CNF, DACNF, and D-DACNF samples. The samples were diluted to the same concentration (0.1 wt%) and three parallel measurements were taken and averaged.

#### 3.6.8. Surface Charge Measurements

The surface charge densities of the CNF, DACNF, and D-DACNF samples were measured by using a particle charge detector (Mütek PCD-03, Mütek Company, Neckartailfingen, Germany). We accurately pipetted 10 mL of 0.1 wt% sample suspension into the PCD measurement bath. A cationic standard solution (poly-DADMAC, 0.001 N) was added dropwise to neutralize the sample charge, and the titration was halted when the potential reached 0 mV. The volume of polydimethyl diallyl ammonium chloride consumed was recorded. Each sample was measured three times in parallel. The surface charge C_1_ (eq/L) of the sample can be calculated by using Equation (5):

(5) $$C_{1\;}=\frac{C_{2}\cdot V_{2}}{V_{1}}$$

where *C*_2_ is surface charge of standard solution (eq/L), *V*_2_ is the consumption of the standard solution (mL), and *V*_1_ is the volume of the sample (mL).

#### 3.6.9. Settling Performance 

The CNF, DACNF, and D-DACNF samples were prepared as suspensions with identical concentrations and subsequently transferred into glass vials, respectively. Photographs were captured by using a camera after the samples had been allowed to stand for 24 h.

#### 3.6.10. Bound Water of CNFs and DACNFs

A differential scanning calorimeter (DSC) (214 Polymer, Netzsch, Germany) was used to determine the bound water (BW) content in the C-CNF and C-DACNF samples [21]. The continuous melting method of DSC was used. First, approximately 5–15 mg of each sample was placed in a sealed crucible. Then, the sample was initially cooled to −30 °C at a rate of 5 °C/min, held for 10 min, and then subsequently warmed to 10 °C with a speed of 1 °C/min. The freezing bound water (FBW) contents and free water contents of the C-CNF and C-DACNF samples were determined from the integral areas of the respective FBW and free water peaks in the DSC heat flow/time curves. The non-freezing bound water (NFBW) content is equal to the total water content of the sample minus the sum of the FBW content and the free water content.

#### 3.6.11. Pore Detection of Concentrated CNFs and DACNFs 

The water within the pores of the concentrated samples (C-CNFs and C-DACNFs) could be measured by using the isothermal step melting method of DSC [21]. About 10 mg of each sample was placed in a sealed crucible. The temperature of the concentrated sample decreased to −30 °C, with a cooling rate of 5 °C/min. To achieve thorough freezing, the sample was maintained at this temperature for a duration of 10 min. Then, the sample temperature was sequentially raised to −20, −15, −10, −6, and −4 °C at a heating rate of 1 °C/min, and it was maintained for 6 min upon reaching the set temperature. After that, the temperature increased successively at a rate of 1 °C/min to −2, −1.5, −1, −0.5, and −0.1 °C. The hold time was 10, 20, 20, 15, and 15 min when each corresponding temperature point was reached. The FBW contents in concentrated sample (C-CNFs and C-DACNFs) pores of varying size were determined by individually integrating the endothermic peak at each melting temperature in the DSC heat flow/time curve. The FBW content unit (g/g) represents the mass of FBW per gram of wet sample (g). The detailed calculation procedure can be referred to in the previous literature [21,51]. The Gibbs–Thomson Equation (6) was used to determine the size of the pore associated with each melting temperature in the concentrated samples (C-CNFs and C-DACNFs).

(6) $$△T=T_{0}-T_{m}=\frac{-4T_{0}\cdot \gamma_{1s}cos\theta }{D\rho H_{f}}$$

where *T*_0_ represents the melting temperature of free water and its value is 273.15 K. *γ*_1*s*_ is the surface energy at the ice–water interface (12.1 mJ/m^2^). *ρ* is the density of FBW (1000 kg/m^3^). *H_f_* indicates the specific heat of fusion of FBW (334 J/g). *D* indicates the diameter of the pore. Δ*T* is the change in melting temperature.

#### 3.6.12. Field Emission Scanning Electron Microscopy (FESEM)

An SEM (Merlin, Zeiss, Germany) was used to analyze the surface morphologies and cross-sectional structures of films at an acceleration voltage of 5 kV. The films were fractured by immersing them in liquid nitrogen to obtain their cross-sections. Before observation, the samples had to be coated with Au.

#### 3.6.13. Surface Roughness of Films

A 3D Optical Profiler (RTEC UP Dual Model, San Jose, CA, USA) was used to examine the surface morphologies of the CNF, DACNF, and D-DACNF films, followed by roughness calculation using Gxyddion software, Version 2.63. The reported values for each film were determined by averaging three test results.

#### 3.6.14. Mechanical Properties of Films

The mechanical properties of the CNF, DACNF, and D-DACNF films were tested by using a testing machine (Instron 5565, Boston, MA, USA) equipped with a 500 N load cell. The films were cut into rectangular shapes (5 mm × 30 mm) for testing, and the test speed was set to 5 mm/min. The reported values for each film were determined by averaging three test results.

#### 3.6.15. UV Transmittance of Films

The transmittance of the films (30 g/m^2^) was tested by using a UV spectrophotometer (UV-2600, Shimadzu, Kyoto, Japan). The wavelength range of the test was 400 to 800 nm. The reported values for each film were determined by averaging three test results.

#### 3.6.16. Water Absorption Properties of Films

The size of the films was reduced to the same dimensions. The films were immersed in water for a specific time (5, 15, 30, 45, 80, 120 min), respectively. Then, water on the surface of the films was absorbed using filter paper before weighing. Subsequently, the mass of the films at each time was weighed. The reported values for each film were determined by averaging three test results. The swelling ratios (SRs) of the films was calculated by using Equation (7):

(7) $$\mathrm{SR}(\%)=\frac{W_{e}{-W}_{0}}{W_{0}}\times 100\;$$

where *W_e_* refers to the weight of the films following immersion, and *W*_0_ represents the weight of the films after drying.

#### 3.6.17. Static Water Contact Angle (WCA) of the Films

An ZJ-7000 water contact angle (WCA)-measuring meter was employed for static WCA measurements of the films. The films were initially positioned on the platform, and then a 5 μL droplet of deionized water was dispensed onto the surface of films using an auto-piston syringe. A photograph of the droplet on each film was captured by using the high-speed camera of the device. The reported water contact angle measurements were values obtained at 20 s of adding droplets to the films.

#### 3.6.18. Water Vapor Permeability and Oxygen Permeability of Films 

A water vapor transmission rate tester (PERMATRAN-W^®^Model 3/34, MOCON, Minneapolis, MN, USA) was used to measure the water vapor transmission rate (WVTR) of the films at 23 °C and 50% RH. An oxygen transmission rate tester (OX-TRAN^®^Model, MOCON, Minneapolis, MN, USA) was employed to measure the oxygen transmission rate (OTR) of the films at 23 °C and 0% RH. The sample films had an area of 50.24 cm^2^ in both the WVTR and OTR tests. The reported values are the average of three measurements taken for each film.

## 4. Conclusions

In this study, the differences in the properties between CNFs isolated from cotton linter fibers and wood fibers were investigated. Subsequently, DACNFs were prepared by periodate oxidation treatment and their morphological structure, physicochemical properties, and structural changes after concentration and dewatering were investigated. In addition, the properties of the films prepared from both DACNFs after concentration and redispersion were analyzed. The results showed that the cotton fiber-based CNFs had a slightly larger diameter (69.51 nm vs. 52.03 nm), higher crystallinity (73.44% vs. 62.89%), but lower water retention (311.38% vs. 427.95%) than that of the wood fiber-based CNFs. The periodate oxidation treatment was beneficial to reducing the size of the DACNFs, the mean diameter of the cotton fiber-based CNFs decreased from 69.51 nm to 38.81 nm, and the mean diameter of the wood fiber-based CNFs decreased from 52.03 nm to 11.93 nm. Due to the high crystallinity of the cotton fiber-based DACNFs, there was no significance difference between the concentrated cotton fiber-based DACNFs and the concentrated cotton fiber-based CNFs. This result indicates that the structural properties of the cotton fiber-based DACNFs were hardly affected by the concentration process. The redispersed cotton fiber-based DACNF films were superior to the redispersed wood fiber-based DACNF films in terms of gas permeability, optical properties, and morphological properties. This study provides a possibility to explore the application of cotton fiber-based CNFs and their derivatives in large-scale production, especially in the field of packaging film materials. 

## Figures and Tables

**Table 1:** Properties of non-concentrated and redispersed cotton fiber-based DACNFs and wood fiber-based DACNFs.

Sample	CNF_CF_	DACNF_CF_	D-DACNF_CF_	CNF_BH_	DACNF_BH_	D-DACNF_BH_
WRV (%)	311.38 ± 0.61	362.31 ± 7.90	354.44 ± 7.53	427.95 ± 7.14	521.15 ± 7.09	473.02 ± 7.21
SSA (m^2^/g)	249.26 ± 0.55	379.64 ± 2.75	292.09 ± 4.31	658.82 ± 0.28	793.27 ± 4.27	192.06 ± 11.56
CrI (%)	73.44	70.33	70.73	62.89	54.46	54.53
D_hkl_ (nm)	4.59	4.45	4.78	3.10	2.99	2.88
Zeta potential (mV)	−14.96 ± 0.81	−15.82 ± 0.31	−14.10 ± 1.17	−19.84 ± 0.33	−23.97 ± 0.84	−17.19 ± 0.14
Surface charge density (eq/g)	(9.59 ± 0.07) × 10^−5^	(4.43 ± 0.04) × 10^−5^	(1.03 ± 0.01) × 10^−5^	(2.75 ± 0.22) × 10^−4^	(5.30 ± 0.02) × 10^−5^	(0.86 ± 0.01) × 10^−5^

**Table 2:** Chemical compositions of cotton linter fiber and BHKP.

Pulp	Chemical Composition (%)		
	Klason Lignin	Glucose	Hemicelluloses
Cotton linter fiber	0.45 ± 0.03	94.15 ± 0.12	0.83 ± 0.01
BHKP	0.74 ± 0.08	74.70 ± 0.64	14.90 ± 0.32

## Data Availability

The data presented in this study are available on request from the corresponding author.

## References

[1] Jechan L., Soosan K., Siming Y., Young-Kwon P. (2023). Bioenergy generation from thermochemical conversion of lignocellulosic biomass-based integrated renewable energy systems. Renew. Sustain. Energy Rev..

[2] Wang S., Song T., Qi H., Xiang Z. (2021). Exceeding High Concentration Limits of Aqueous Dispersion of Carbon Nanotubes Assisted by Nanoscale Xylan Hydrate Crystals. Chem. Eng. J..

[3] Sixta H., Potthast A., Krotschek A.W. (2006). Chemical Pulping Processe: Sections 4.1–4.2.5. Handbook of Pulp.

[4] Pennells J., Godwin I.D., Amiralian N., Martin D.J. (2019). Trends in the production of cellulose nanofibers from non-wood sources. Cellulose.

[5] Jiang J., Zhu Y., Jiang F. (2021). Sustainable isolation of nanocellulose from cellulose and lignocellulosic feedstocks: Recent progress and perspectives. Carbohydr. Polym..

[6] Sixta H., Süss H.-U., Potthast A., Schwanninger M., Krotscheck A.W. (2006). Pulp Bleaching: Sections 7.1–7.3.5. Handbook of Pulp.

[7] Gümüskaya E., Usta M., Kirci H. (2003). The effects of various pulping conditions on crystalline structure of cellulose in cotton linters. Polym. Degrad. Stab..

[8] Zhang S., Han Y., Wang G., Feng L., Lei Y., Wang Z., Xiong S., Yang B., Du W., Zhi X. (2023). Long-term assessments of cotton fiber quality in response to plant population density: Reconciling fiber quality and its temporal stability. Ind. Crops Prod..

[9] Maiti S., Jayaramudu J., Das K., Reddy S.M., Sadiku R., Ray S.S., Liu D. (2013). Preparation and characterization of nano-cellulose with new shape from different precursor. Carbohydr. Polym..

[10] Hemmati F., Jafari S.M., Taheri R.A. (2019). Optimization of homogenization-sonication technique for the production of cellulose nanocrystals from cotton linter. Int. J. Biol. Macromol..

[11] Kim U.J., Kuga S. (2000). Reactive interaction of aromatic amines with dialdehyde cellulose gel. Cellulose.

[12] Sun B., Hou Q., Liu Z., Ni Y. (2015). Sodium periodate oxidation of cellulose nanocrystal and its application as a paper wet strength additive. Cellulose.

[13] Kim U.-J., Kimura S., Wada M. (2019). Highly enhanced adsorption of Congo red onto dialdehyde cellulose-crosslinked cellulose-chitosan foam. Carbohydr. Polym..

[14] Silva Gomes A., Vitória Guimarães Leal M., Roefero Tolosa G., Camargo Cabrera F., Dognani G., Eloízo Job A. (2023). Cationic dialdehyde cellulose microfibers for efficient removal of eriochrome black T from aqueous solution. Bioresour. Technol..

[15] Yao M., Wang Z., Liu Y., Yang G., Chen J. (2019). Preparation of dialdehyde cellulose graftead graphene oxide composite and its adsorption behavior for heavy metals from aqueous solution. Carbohydr. Polym..

[16] Lei Z., Gao W., Zeng J., Wang B., Xu J. (2019). The mechanism of Cu (II) adsorption onto 2,3-dialdehyde nano-fibrillated celluloses. Carbohydr. Polym..

[17] Koprivica S., Siller M., Hosoya T., Roggenstein W., Rosenau T., Potthast A. (2016). Regeneration of Aqueous Periodate Solutions by Ozone Treatment: A Sustainable Approach for Dialdehyde Cellulose Production. ChemSusChem.

[18] Isogai A. (2020). Emerging Nanocellulose Technologies: Recent Developments. Adv. Mater..

[19] Liu H., Tu Q., Huang L., Gao W., Zeng J., Wang B., Xu J., Wang Z. (2021). Characteristics of concentrated lignocellulosic nanofibril suspensions. Cellulose.

[20] Yuko O., Miyuki T., Akira I. (2022). Characterization of solid-state structures, molar masses, and microfibril structures of cellulose in never-dried cotton fibers and ramie bast fibers. Cellulose.

[21] Hefang L., Qiyuan T., Luyao H., Wenhua G., Jinsong Z., Bin W., Jinpeng L., Jun X. (2023). Characteristics of concentrated cellulose nanofibrils measured by differential scanning calorimetry. Cellulose.

[22] Jiang X., Yang Z., Peng Y., Han B., Li Z., Li X., Liu W. (2015). Preparation, characterization and feasibility study of dialdehyde carboxymethyl cellulose as a novel crosslinking reagent. Carbohydr. Polym..

[23] Agarwal U.P., Reiner R.S., Ralph S.A., Catchmark J., Chi K., Foster E.J., Hunt C.G., Baez C., Ibach R.E., Hirth K.C. (2021). Characterization of the supramolecular structures of cellulose nanocrystals of different origins. Cellulose.

[24] Khandoker Samaher S., Nitesh Kumar K., Ashiqur R.M., Hasan J., Youssef H., Stephen J.E., Alfred D.F., Lokendra P., Lucian A.L. (2023). Comparison and assessment of methods for cellulose crystallinity determination. Chem. Soc. Rev..

[25] Duchemin B. (2017). Size, shape, orientation and crystallinity of cellulose Iβ by X-ray powder diffraction using a free spreadsheet program. Cellulose.

[26] Cao X., Wang Y., Chen H., Hu J., Cui L. (2021). Preparation of different morphologies cellulose nanocrystals from waste cotton fibers and its effect on PLLA/PDLA composites films. Compos. Part B Eng..

[27] Errokh A., Magnin A., Putaux J.-L., Boufi S. (2018). Morphology of the nanocellulose produced by periodate oxidation and reductive treatment of cellulose fibers. Cellulose.

[28] Dias M.C., Zidanes U.L., Martins C.C.N., de Oliveira A.L.M., Damásio R.A.P., de Resende J.V., de Barros Vilas Boas E.V., Belgacem M.N., Tonoli G.H.D., Ferreira S.R. (2022). Influence of hemicellulose content and cellulose crystal change on cellulose nanofibers properties. Int. J. Biol. Macromol..

[29] Gu F., Wang W., Cai Z., Xue F., Jin Y., Zhu J.Y. (2018). Water retention value for characterizing fibrillation degree of cellulosic fibers at micro and nanometer scales. Cellulose.

[30] Ding Q., Zeng J., Wang B., Tang D., Chen K., Gao W. (2018). Effect of Nanocellulose Fiber Hornification on Water Fraction Characteristics and Hydroxyl Accessibility during Dehydration. Carbohydr. Polym..

[31] Sulaeva I., Klinger K.M., Amer H., Henniges U., Rosenau T., Potthast A. (2015). Determination of molar mass distributions of highly oxidized dialdehyde cellulose by size exclusion chromatography and asymmetric flow field-flow fractionation. Cellulose.

[32] Chen W., Abe K., Uetani K., Yu H., Liu Y., Yano H. (2014). Individual cotton cellulose nanofibers: Pretreatment and fibrillation technique. Cellulose.

[33] Zhang Y., Deng W., Liu C., Yan F., Wu M., Cui Q., Willför S., Xu C., Li B. (2023). Preparation of Antibacterial Dialdehyde Nanocellulose Using LiBr·3H_2_O Non-Dissolving Pretreatment Promoted Periodate Oxidation. ACS Sustain. Chem. Eng..

[34] Martínez-Sanz M., Pettolino F., Flanagan B., Gidley M.J., Gilbert E.P. (2017). Structure of cellulose microfibrils in mature cotton fibres. Carbohydr. Polym..

[35] Liyanage S., Abidi N. (2019). Molecular weight and organization of cellulose at different stages of cotton fiber development. Text. Res. J..

[36] Wang H., Chen C., Fang L., Li S., Chen N., Pang J., Li D. (2018). Effect of delignification technique on the ease of fibrillation of cellulose II nanofibers from wood. Cellulose.

[37] Zhang X., Li P., Zeng J., Li J., Wang B., Gao W., Xu J., Chen K. (2023). Dynamic Covalent Bond Enabled Strong Bio-based Polyimide Materials with Thermally-driven Adaptivity, Healability and Recycling. Chem. Eng. J..

[38] Chu Y., Sun Y., Wu W., Xiao H. (2020). Dispersion Properties of Nanocellulose: A Review. Carbohydr. Polym..

[39] Jing S., Wu L., Siciliano A.P., Chen C., Li T., Hu L. (2023). The Critical Roles of Water in the Processing, Structure, and Properties of Nanocellulose. ACS Nano.

[40] Solhi L., Guccini V., Heise K., Solala I., Niinivaara E., Xu W., Mihhels K., Kröger M., Meng Z., Wohlert J. (2023). Understanding Nanocellulose–Water Interactions: Turning a Detriment into an Asset. Chem. Rev..

[41] Hatakeyama T., Nakamura K., Hatakeyama H. (1988). Determination of bound water content in polymers by DTA, DSC and TG. Thermochim. Acta.

[42] Hatakeyama T., Iijima M., Hatakeyama H. (2016). Role of bound water on structural change of water insoluble polysaccharides. Food Hydrocoll..

[43] Lin G., Xu J., Wu M., Sun Q., Zhu S., Wang B., Zhang W. (2023). Cellulose Nanofibril/Talc Composite Films with Excellent Barrier Properties by Alternate Hierarchical Method. ACS Appl. Polym. Mater..

[44] Hou Y., Wang X., Yang J., Zhu R., Zhang Z., Li Y. (2018). Development and biocompatibility evaluation of biodegradable bacterial cellulose as a novel peripheral nerve scaffold. J. Biomed. Mater. Res. Part A.

[45] Ismail M.F., Islam M.A., Khorshidi B., Tehrani-Bagha A., Sadrzadeh M. (2021). Surface characterization of thin-film composite membranes using contact angle technique: Review of quantification strategies and applications. Adv. Colloid Interface Sci..

[46] López Durán V., Hellwig J., Larsson P.T., Wågberg L., Larsson P.A. (2018). Effect of Chemical Functionality on the Mechanical and Barrier Performance of Nanocellulose Films. ACS Appl. Energy Mater..

[47] Guo L., Li M., Xu Q., Jin L., Wang Y. (2022). Bio-based films with high antioxidant and improved water-resistant properties from cellulose nanofibres and lignin nanoparticles. Int. J. Biol. Macromol..

[48] Sluiter A., Hames B., Ruiz R., Scarlata C., Sluiter J., Templeton D., Nrel D. (2011). Determination of structural carbohydrates and lignin in biomass determination of structural carbohydrates and lignin in biomass. Technical Report: NREL/TP-510-42618.

[49] Wada M., Okano T., Sugiyama J. (1997). Synchrotron-radiated X-ray and neutron diffraction study of native cellulose. Cellulose.

[50] Kaewprasit C., Hequet E., Abidi N., Gourlot J.P. (1998). Application of methylene blue adsorption to cotton fiber specific surface area measurement: Part I. Methodology. J. Cotton Sci..

[51] Park S., Venditti R.A., Jameel H., Pawlak J.J. (2006). Changes in pore size distribution during the drying of cellulose fibers as measured by differential scanning calorimetry. Carbohydr. Polym..

